# The Preservation of Food in Ancient and Modern Times
*The Pantropheon, or History of Food and Its Preparations from the Earliest Ages of the World. By A. Soyer. London: Simpkin and Marshall. 1855.Conservation des Substances Alimentaires. Par M. Poggiale. *Gazette Médicale de Paris*, Nov. 1st and 8th, 1856.Preservation of Alimentary Substances. By M. Poggiale, *Gazette Médicale de Paris*.On the Composition of Food, and how it is Adulterated. By W. Marcet, M.D., F.C.S. London: Churchill. 1856.


**Published:** 1857-01

**Authors:** 


### THE PRESERVATION OF FOOD IN ANCIENT AND MODERN TIMES.*

Surely of all the sumptuous dishes that ever came from the hands of mortal cook, from Apicius downwards, there has not been one of a literary character so remarkable as M. Soyer's *Pantropheon*, a book of elegant cast, full of the most exquisite plates, not of trussed rabbits or scored oxen, but of goddesses, antique dishes, banquets, portraits of distinguished individuals, from Epicurus to M. Soyer himself; to add nothing about stewpans, stockpots, winecups, and spoons. Then, as to matter, such classical histories, so voluminous indeed as to require a table of from two to three thousand references from all the great works of antiquity. A truly tremendous scholar is this M. Soyer!

However, with this note of admiration we must end our compliments and introduction. We have to consider a special subject, and we hope to obtain from the *Pantropheon* one or two easily digestible crumbs of information.

The attempt to preserve animal food is of ancient date. It sprang naturally, we presume, from a desire to make provision for days and seasons when it should be impossible to obtain the first fruits of the earth. The drying and preservation of corn and fruits would be the first step in this direction. Then, perchance, followed the invention of cheese, which M. Soyer, following Justin, declares to have

---

* The Pantropheon, or History of Food and Its Preparations from the Earliest Ages of the World. By A. Soyer. London: Simpkin and Marshall. 1855.

Conservation des Substances Alimentaires. Par M. Poggiale. *Gazette Médicale de Paris*, Nov. 1st and 8th, 1856.

Preservation of Alimentary Substances. By M. Poggiale. *Gazette Médicale de Paris.*

On the Composition of Food, and how it is Adulterated. By W. Marcet, M.D., F.C.S. London: Churchill. 1856.

been invented by no less a personage than Aristæus, a demi-god, the son of Apollo himself, and the king of Arcadia.

"The most ancient form of preservation of animal foods," says Poggiale, "is probably that of desiccation, for we know the practice of drying meat in the sun is common all over America; and M. Boussingault has spoken to some negroes of Choco who have never seen an ox, but only know its muscular dried flesh. This learned chemist, indeed, supported himself on dried meats for nearly three years, which he spent in the mines of la Vega during his excursion to the gold and platina washings." However, the meat thus dried is very hard, and affords an unsavoury and indigestible food.

But although the process of desiccation may have been the most primitive and wide-spread, as derived from the observation of the simplest natural phenomena, the mode of preserving by means of salt is of very remote, if not of equally remote date. "Thus the Roman butchers sold both fresh and salt meat. Their mode of preparing the salt meat being as follows. The animals they intended to salt they kept from drinking anything on the eve of the day before killing. They boned the meat and sprinkled it lightly with pounded salt; then, having well dried off all dampness, they sprinkled some more salt, and placed the pieces, so as not to touch each other, in vessels which had been used for oil or vinegar. They poured sweet wine over, covered the whole with straw, and strewed snow all around, in order to make the meat better and more tender. When the cook wanted to extract the salt, he first boiled the meat well in milk, and afterwards in soft water."

The importance attached to the use of salt previous to Roman times is shown by the fact that the Greeks placed this substance in the list of things which were consecrated to the gods; so that it was considered a misfortune to spill salt, and impious to forget to place salt-cellars on the tables, or to go to sleep before their removal.

But there is yet another ancient mode of preserving food, namely, the exclusion of the preserved substances from the air. Thus Apicius recommends, for the preservation of vegetables, that they should be chosen before they are perfectly ripe, placed in a vessel covered with pitch, and sealed hermetically. In like manner, the Roman butchers preserved the flesh of various animals without salt. They covered each piece of meat with honey, put it in a vessel hermetically sealed, and hung it in a cool place—an operation which was said to succeed well. Apicius also recommends, for the preservation of pork, a process of a similar character. The pieces are to be entirely covered with paste composed of salt, vinegar, and honey, and placed in vessels carefully closed,

Lastly, the exposure of alimentary substances to certain gaseous preservatives is a plan of ancient times. Common wood smoke is one of the preservatives which has been thus employed both in past ages and in modern times. In England a very old custom of preserving fruits, gooseberries especially, consists in filling a vessel with the fruit, then burning in the neck of the vessel a piece of sulphur and corking up securely while yet the sulphur is burning. By these means a quantity of sulphurous acid gas is generated, and is diffused through and detained in the bottle, while at the same time a portion at least of the free oxygen surrounding the fruit is removed.

In these four processes are included the practical parts of all modern plans for the preservation of alimentary substances. In order to understand the mode in which they preserve, it is necessary to know in what the process of putrefaction consists. This is well described for us by M. Poggiale.

After death, the vital force no longer opposing itself to the chemical or physical forces, the organic matters present those particular phenomena to which we have given the name of fermentation. The organic elements then form most simple combinations, and this transformation acts by reactions which have no analogy with the ordinary chemical decompositions. No one is ignorant of the fact that organic matters do not putrefy except under the influence of water, of oxygen, and of a suitable temperature, and that if they are withdrawn from the action of one of these agents they do not ferment. All the processes which have been conceived for the preservation of foods are based therefore on these elements—expulsion of the water, withdrawal of the air, and a low temperature; these are the usual means which have been put in practice under a thousand forms.

The subjoined abridgment of M. Poggiale's very complete memoir will give a good idea of the nature of the processes which are now used for the purpose of preserving meat, milk, and vegetables.

### PRESERVATION OF MEATS.

The process of Appert consists in enclosing in cylindrical glass or earthenware bottles the aliments, closing the mouth of the vases with great care, and submitting them during a longer or shorter time to the action of boiling water. Cases of tinned iron are now preferred, because there is no risk of breakage, and they can be closed more securely. The cooked meat, still boiling, is placed in the cases, and pressed down moderately so as to fill the vessel. The circular cover is then soldered to the tin, leaving a small opening in the middle. The vessel is then filled with the juice of the meat or the broth, and a small disk of tinned iron is soldered over the opening. Several cases filled in this manner are placed in a case heated by steam, or in a copper containing boiling water. When they are withdrawn, the covers are convex, but they soon become flat and even concave. This denotes complete success. If the cover remains convex, the preservation of the meat is not ensured. This process consists, then, in destroying the influence of the oxygen of the air. The oxygen which remains enclosed with the meat combines with the organic matters, and can no longer excite fermentation. According to Gay-Lussac, the heat should be sufficiently prolonged to destroy or solidify the substance which has absorbed the oxygen, and which would produce fermentation. It is of course necessary that the vessels be closed accurately, so that the air cannot penetrate into them.

Experience of more than forty years has shewn the success of the process. These preserved meats have been taken across the equator, brought back to London, and sent out again to the polar regions. Sixteen years afterwards the meat was of the best flavour. The campaign in the East has given a new importance to Appert's method. The boxes of meats prepared by this process, and destined for the army in the Crimea, were of excellent quality.

M. Fastier has perfected Appert's process, by almost entirely expelling the air enclosed in the aliments. He introduces the meat raw into the tinned iron boxes; solders the cover, which is pierced with a small opening, then heats the vessels in a water-bath containing common salt or the chloride of calcium so that the temperature is raised to 230° Fahr., and ebullition takes place within the boxes. The steam, and with it nearly all the air, then rushes out from the aperture. The vessels are now completely filled; and the opening is soldered. The boxes are surrounded now with cold water, by which a vacuum is formed in their interior. They are heated a second time; the aperture is unstopped; and when the air and steam have gone off, they are closed anew.

Meats preserved by this process have been found at the end of a year or two in a perfect state of preservation, of excellent quality, and of agreeable flavour. The Commission of Military Stores has remarked on the superiority of M. Fastier's productions over those of Appert.

M. de Lignac has also modified the process, by introducing the meat in large pieces instead of its being cut up. M. de Lignac also cuts the meat into small pieces, heating it in a stove to the temperature of 104° Fahr., so as to remove about two-thirds of the water, submits it to energetic pressure in a tube of tinned copper, and then completes the process by the ordinary means in closed boxes. M. Poggiale speaks favourably of the results of this process.

M. Cellier employs the following process for the preparation of powdered meat. The bones and the greater part of the fat are taken out; then the meat is cut into strips, which are dried in a stove at the temperature of 122° to 132° Fahr., and reduced to powder by a rasp and pestles. Two parts of powdered meat are equivalent to about four of lean meat, and to six of meat containing the bones and fat. The meat dried by M. Cellier is in the form of a coarse powder, and is capable of long preservation if it be sheltered from the damp and in suitable receptacles, but it is indispensable to deprive it of a large portion of the fat. The small bulk of the meat prepared by M. Cellier's method points to its convenience under certain circumstances of war: but it should be employed only in special cases. Its appearance is not very agreeable; and it has the inconvenience of quickly passing the digestive canal. It is doubtful if young and robust men would be nourished by this powdered meat.

For salting meat the English process, without contradiction, says M. Poggiale, furnishes the best results. It consists in putting pieces of meat in contact with a mixture of saltpetre, common salt, and sugar, and renewing this operation often. But the ordinary processes of salting cause contraction of the muscular fibres, and make the meat hard and often difficult of digestion. Common salt deteriorates the nutritive value of the meat. The salt added takes up from the meat a considerable quantity of water, which carries with it a certain amount of nutritive substance. According to M. Liebig, two parts out of three of meat may, by the action of the salt, become unfit to sustain the vital functions.

Another method of preservation consists in keeping the meat from the action of the air by a covering of gelatine. In this process the meat is covered with a jelly prepared by boiling the tendinous parts of animals for a long time, and by concentrating the dissolved parts. A small quantity of sugar, of gum arabic, and of brandy, is now added to it, and the pieces of meat, suitably cut, are plunged into this liquid, at a temperature of about 150° or 160° Fahr., and are then suspended in the open air by a hook. This operation is repeated a second time. The next day the envelope is firm.

A committee, composed of MM. Michel Lévy, Laperlier, and Poggiale, ascertained that these meats dry little by little without experiencing any change when they are kept hanging and in the free air, but that the alteration of the covering, and friction during transportation, expose the flesh to the action of the air, and consequently bring on putrefaction. Meats enveloped in gelatine were enclosed in cases, some of which were placed in the military stores, and the remainder sent to Constantinople with orders to send them back to France. On their return, the several samples were completely spoiled.

Another process, proposed by a former professor of the University of France, consists in preserving animal matters, fruit, and vegetables, in sulphurous acid. The alimentary substances are placed in wooden boxes lined with zinc, and having a double bottom of zinc into which chloride of calcium is introduced through a large hole. The upper part of the box has a plate of glass fixed with mastic or a sheet of zinc soldered. Sulphurous acid is introduced into the box and drives out the air through an opening in the upper part. When the acid begins to escape into the atmosphere, both holes are closed with great care. The sulphurous acid without doubt suspends the action of the ferment, which produces the putrefaction of the organic substances. The experiments which the inventor made at the Val-de-Grâce completely failed: all the foods which he attempted to preserve in this way were spoiled at the end of a few days. Experiments, however, which are now made with sulphurous acid, seem to promise good results.

It has been proposed to preserve meats in water containing a tenth of sulphuric acid. The meat is first boned and washed with cold water, and then plunged for an hour into the acid liquor. It is then carefully barreled, and the barrels are filled with water containing a hundredth part of sulphuric acid, and closed as hermetically as possible. According to the inventor, the meat thus preserved appears perfectly fresh; it is juicy, and the broth and the gravy yielded by it are of the best flavour. Unfortunately, the experiments made by a committee at the Val-de-Grâce have not confirmed these results. At the end of fifteen days the meat was putrid.

A number of attempts have been made to fabricate and preserve meat biscuit and the extract of meat. An American, Gail Bordeu, prepared a biscuit some years since by mixing flour with cooked meat and the liquor in which it had been boiled. The inventor affirmed that the biscuit might take the place of bread and meat, and that about five and a half drachms avoirdupois would suffice to nourish a workman for twenty-four hours, and perfectly maintain his strength. These hopes have not been realised.

M. Callamand prepares a biscuit with a mixture of fifty parts of beef, one hundred of wheaten flour, and ten of vegetables. After the meat has been washed in water acidulated with vinegar, it is cooked with the vegetables for eight hours, and the liquor concentrated. The bones, tendons, cartilages, etc., are taken out, and only the muscles and fat left; the meat is heated afresh; powdered sugar-candy is added in the proportion of one part to 640 of the meat, flour, and vegetables originally used. It is then kneaded together with the flour. The dough is formed into biscuits, and baked about an hour and a half. According to M. Poggiale, M. Callamand's biscuit has a brown colour, and a very distinct smell and taste of fat. It easily crumbles, and does not appear suitable for a long voyage. M. Poggiale found a considerable proportion of fatty matter in the cakes, but less of nitrogenous principles than in the hard corn biscuits prepared at the military stores. M. Callamand's biscuit, and all analogous products, have been rejected by the ministry of war, on account of the uncertainty attending their manufacture, and their deficiency in nutritive properties.

*Extract of Meat.*—Parmentier has recommended the use of extract of meat in the army. He considers that, mixed with a little wine, it supports the strength of the wounded, and enables them to endure the fatigue of a long removal. M. Liebig says that, in countries where beef and mutton are plentiful and cheap, as in Podolia, Buenos Ayres, Mexico, and Australia, large quantities of extract of meat might be prepared, and imported to Europe. To prepare this preserve, M. Bellat, an apothecary of Paris, takes meat as fresh as possible; removes the fatty, tendinous, and membranous parts; divides it into very thin pieces, and then places them in an apparatus where fresh water is allowed to percolate until the fluid passes colourless and insipid. The products of this operation are then set aside. The meat is then placed in tubs heated by steam, and hermetically closed by screwed covers, furnished with a service-pipe with a check valve. Its own weight of water is then added, with a quantity of bones. The whole is left to digest during six hours, at the temperature of 194° Fahr., the meat being agitated. It is then submitted to the action of a hydraulic press, and mixed with water and cooked vegetables. The hot solutions are mixed with liquors prepared cold, and heated in evaporating coppers to coagulate the blood; they are then rapidly filtered. These liquids are evaporated in an apparatus to the consistence of very thick honey. The extract of gravy thus obtained is placed in tin boxes, and fastened down after Appert's method. By this process, according to M. Poggiale, the products are not submitted to any treatment likely to induce change in the nature of the broth. This extract must not be confounded with the products known under the name of broth tablets (*tablettes de bouillon*). These preparations contain a considerable quantity of gelatine, furnished by the bones, cartilages, and tendons. M. Bellat's extract of meat is in the form of a yellowish brown mass, rather soft, very soluble in water, possessing smell, taste, and all the properties of meat broth. By dissolving some in boiling water and adding common salt, a savoury broth is obtained, having all the characteristics of good broth prepared from fresh meat. M. Poggiale is of opinion that the richness of this extract in nitrogenous principles, the ease with which it is converted into a broth of excellent quality, its easy transport and preservation, recommend it for the alimentation of troops, and above all for the use of ambulances and hospitals.

A Dr. L. has prepared broth tablets, which have some analogy with M. Bellat's, but which are inferior in many respects. These tablets have an agreeable taste, a reddish brown colour, are not entirely soluble in water, are perfectly preserved in contact with the air, but they contain a considerable proportion of gelatine.

### PRESERVATION OF MILK.

Among the processes which have been proposed in recent years for the preservation of milk, those of MM. de Lignac and Mabru have received the sanction of experience; and, in 1855, the Academy of Sciences adjudged them a prize.

M. de Lignac evaporates the milk with a water-bath, in copper dishes which contain a layer of only four-tenths of an inch in depth, and adds 60 parts of sugar to 1000 of milk.

The liquid is then continually agitated, until it is reduced to a fifth of its volume. It is then put into tin boxes, which are heated in a water-bath to the temperature of 221° Fahr. At the end of half an hour, the opening which gave passage to the air and steam is closed with solder. The substance contained in the boxes is yellowish, sweet, doughy, easily soluble in water, and makes a liquid which has all the characteristics of milk, if it has not been too much sweetened. When it is required for use, four parts of water, which is the quantity which has been removed by evaporation, are added to one part of concentrated milk. The Commission of Military Substances has submitted this preparation of M. de Lignac to careful examination: it has constantly shown an irreproachable taste and smell.

M. Mabru's process consists in putting the liquid into metallic bottles, terminated in the upper part by a vertical leaden tube, which communicates with a reservoir also containing milk. All parts of the apparatus are entirely filled with milk. The bottles are then placed, in number about twelve or fifteen, in a large closed vessel, to the inside of which steam is conveyed. The milk is then heated to about 170° Fahr., and, in consequence of dilatation, a part of the liquid rises in the upper reservoir, where it finds shelter from the air by a bed of oil which covers the surface. The air entirely escapes by the vertical tube. At the end of an hour it is cooled to the temperature of about 68° Fahr. The volume of milk diminishes by cooling, but it fills the bottle, as well as the tube which surrounds it. The vessel is then hermetically closed by compressing the tube by means of pincers; the tube is then cut above the compressed point, and solder is applied. The milk is thus guarded from the air, and cannot be agitated in the inside of the vessel. This milk, at the end of three years, presented all the characteristics of fresh-drawn warm milk of excellent quality.

### PRESERVATION OF VEGETABLES.

Vegetables may be easily preserved by Appert's method; but the interposing liquids and the vessels which contain these preserves considerably augment the weight and size. On the other hand, the cost of the carriage and the value of the vessels so raise the price of them, that they can only be used by a very small number of consumers.

M. Masson began his researches on the preservation of vegetables in 1844. He had been preceded by MM. Sylvester and Alain, of the school of Grignan, who about 1842

presented to the Horticultural Society of Seine and Oise some dried cabbages.

In 1845, M. Masson obtained a medal of the Royal and Central Agricultural Society. His process at that time differed but little from the old processes; it consisted in exposing the leaves of the cabbages separately on basket-work. In 1850, M. Masson made his first attempt to reduce the size and facilitate the preservation of vegetables by means of the hydraulic press. He reduced the volume of the vegetables about eight-tenths, and gave them the shape of rectangular slabs corresponding to a certain number of rations.

In 1851, M. Chollet added to M. Masson's process a previous scalding in boiling water; a proceeding which is still employed in manufactories for the preparation of certain vegetables. The vegetables prepared by M. Chollet had the smell of hay; and it was necessary, before cooking them, to immerse them in cold water during eight or ten hours, or in tepid water for four hours.

These inconveniences have been removed by a very important improvement, which is the complete cooking of the vegetables before their desiccation, by means of steam at a temperature above 212° Fahr. The vegetables, picked with care, washed, and cut, are cooked in vessels of strong iron by steam coming from a generator. The temperature in the interior of the vessels is from 233° to 240° Fahr; the system of closing, by preventing the escape of the steam, maintains the pressure. The vegetables are cooked in a few minutes. Simmering in boiling water is still used for some vegetables, which require water to dispossess them of some of their acridness; Brussels cabbages, for instance. To this previous cooking the vegetables owe the preservation of their colour and their saccharine principles. The advantages of the employment of steam are its facility of application, the non-interference with the properties of the vegetables, and the coagulation of the vegetable albumen, and consequent prevention of fermentation. Taken from the cooking apparatus, the vegetables are ranged in the drying rooms, a current of air being rapidly carried through at a temperature of 112° to 122° Fahr. Two hours suffice for the drying of spinach, chicory, etc.; three for cabbages, carrots, turnips, etc. Taken from the drying rooms, the vegetables are very crumbly and friable; they are therefore left exposed to the air, that they may gain a little moisture, and thus acquire a certain degree of flexibility. The vegetables are then either compressed or uncompressed. Those which are intended to be compressed are placed in hydraulic presses, and are found to be reduced about eight-tenths of their volume. The tablets thus obtained are square, hard, and heavier than wood. They are enveloped in paper, and put into boxes of zinc or tin.

Analyses show that the dried and compressed vegetables are richer in nitrogenous matters, and have consequently a higher nutritive value, than the same vegetables arrived at their maturity. The following experiments, made on peas, bring forth the influence of maturity on the relative proportions of water and nitrogenous matters.

| | | | Water in 100. | Percentage of nitrogenous matters in dried peas. |
|---|---|---|---|---|
| Exp. I. | Very tender green peas | | 82·25 | 38·35 |
| II. | Ditto | ditto | 83·20 | 38·67 |
| III. | Ditto | ditto | 80·20 | 37·98 |
| Exp. I. | More advanced green peas than the preceding | | 76·14 | 34·17 |
| II. | Ditto | ditto | 75·20 | 34·48 |
| III. | Ditto | ditto | 75·36 | 34·46 |
| Exp. I. | Ripe green peas | | 70·62 | 27·72 |
| II. | Ditto | ditto | 70·49 | 27·43 |
| III. | Ditto | ditto | 70·87 | 27·21 |

M. Chollet furnished to the army, during the campaign in the East, 120,000 rations of preserves a day in winter, and 40,000 in summer. These preserves were composed of tablets of potatoes, and of *julienne* soup, made of carrots, cabbage, and potatoes, of turnips, a small quantity of onions, celery, leeks, and parsnips. In other tablets broad beans were added. These dried vegetables exercised the most happy influence on the health of the French soldiers: the military surgeons have all testified to the hygienic advantages which accrued from the use of the vegetables, especially when the food consisted exclusively of biscuit and salt meat. M. Chollet has tried to associate dried and compressed vegetables with gelatine, by plunging the tablets of vegetables once or several times in a bath of aromatic gelatine containing the juice of meat. He also obtained a series of sizes, according to the thickness of the coats and the number of layers. The soup made from these is more agreeable and alimentary, but it contains too much gelatine.

Another preparation is the semolina of potatoes, proposed by MM. Berncastel and Chollet. To prepare this aliment, the potatoes are cooked, and, after the skin is taken off, they are crushed and transformed into semolina in the usual manner. This product is then dried in a stove: 100 parts of semolina represent about 500 parts of potatoes. The total of nitrogenous matters of potatoes, which is 1·6 per 100, is raised to 8·580 in the semolina. This substance, however, requires animal food to be associated with it. Pea soup, and rich or weak broth, may be prepared with this semolina. These aliments are very agreeable, and easy of digestion. The small volume of this semolina, the facility of cooking it in water, milk, or broth, its easy preservation and transport, recommend it for army use, particularly for ambulances.

Dr. Marcet, in the concluding part of his very useful work *On the Composition of Food*, enters on the subject of preserved food. He enumerates seven ways of preservation; viz., by cold; by exclusion of air; by drying; by salting; by exposure to smoke; by sugar; by vinegar. Regarding the influence of cold in preventing putrefaction, he refers to the facts that frozen meat and fish are transmitted from Archangel to St. Petersburg; and that provisions are sent, packed in ice, from remote parts of England to London. "If," says Dr. Marcet, "food thus preserved has not undergone the slightest decomposition, it is exactly in the same condition as when quite fresh, and consequently as healthy and nutritive."

On the subject of preservation of meat by drying, this author refers to Dr. F. Verdeil's process, which is evidently in its essentials the same as one of those described by M. Poggiale. Dr. Marcet thus comments on the drying process. "Food thus preserved, whether it be animal or vegetable, has the advantage, first, of preserving in a fresh condition, though freely exposed to the atmosphere, for a great number of years; secondly, of being reduced to about one-fifth of its original bulk, from having lost all its water." The vegetables regain their shape and bulk when boiled in water; the soup possesses all the aroma of the food to such an extent, that it is often difficult to notice the difference between it and soups prepared from recently gathered vegetables.

In what has been already written, we have supplied the reader with a knowledge of all the more important plans of preserving foods. If we were asked for a choice of plans, we should certainly go with Dr. Marcet in recommending Verdeil's. In a sanitary point of view, it is a matter of considerable moment to put out of fashion the plan of preserving meats by salt; salted preserves being at once indigestible, and, if long continued too exclusively, the certain cause of some adynamic disorders, such as scurvy.

In glancing at the previously named plans of preserving foods, it will be seen that they are all directed virtually to the end of preventing the action of oxygen on the dead tissue.

It should at the same time be explained, however, that, in order to prevent this oxidation, it is neither necessary to subject the meat itself to any process, nor even to remove it from the presence of oxygen. There are in nature various gaseous agents which, when commingled with oxygen, prevent the action of that gas, by exerting a kind of counter-affinity. These agents, all of which are for this reason preservatives of animal and vegetable structures, are creasote vapour, chloroform vapour, carbonic acid gas, common coal gas, and sulphurous acid. To these others might be added, which are preservative, but are not applicable for foods. It is remarkable that even oxygen itself, when used in the pure form, *i. e.* undiluted with nitrogen, has a certain degree of antiseptic power; and we have ourselves kept a portion of beef in oxygen gas for many months, the meat itself undergoing but little visible change. This, however, is not sufficiently effective. The most effective plans consist, first, in having made an air-tight iron safe. In this the meat must be suspended; and then, if sulphurous acid is to be the preservative, a small lump of burning sulphur must be laid on the floor of the safe, the door being instantly closed, so that the sulphurous fumes may not escape. If chloroform is to be used, a few drops should be dropped simply on the floor of the safe, and so be left, the door again being closed instantly. If carbonic acid gas is used, it will first have to be generated outside, and then be driven into the safe, previously closed and containing the meat, through a stop-cock.

It is unnecessary to extend these descriptions, because the old fashioned sulphur process, and the new fashioned chloroform process, answer all practical purposes, and are by far the simplest and readiest in application. The vapours of these agents, in which the preserving power lies, are innocuous by the time the food comes to table, since they are driven off in cooking. In the year 1851, the writer took occasion to show that the immersion of animal structures in pure nitrogen, in bottles hermetically sealed, was an excellent mode of preservation; and he has since found that the placing of a stick of phosphorus in a closed vessel with animal substances, answers for a time remarkably well, the phosphorus removing the oxygen. He does not, however, at this moment recommend this last named mode for the preservation of edible substances, the effect of the phosphorous vapours on the preserved tissue being unknown.

